# There is more to life than risk avoidance – elderly people’s experiences of falls, fall-injuries and compliant flooring

**DOI:** 10.1080/17482631.2018.1479586

**Published:** 2018-06-05

**Authors:** Johanna Gustavsson, Carolina Jernbro, Finn Nilson

**Affiliations:** Centre for Public Safety, Faculty of Health, Science and Technology, Karlstad University, Karlstad, Sweden

**Keywords:** Injury prevention, impact-absorbing flooring, low-impact flooring, nursing home, residential care, fall injury

## Abstract

**Purpose:** Falls are the most common cause of injury in all ages and are especially difficult to prevent among residential care residents. Compliant flooring that absorbs energy generated within the fall, has been proposed as a measure to prevent fall-injury, however little is known regarding the implementation aspects in clinical settings. The aim of this study is to explore the experiences of falls, the risk of fall-injury, prevention in general and specifically compliant flooring as an injury preventative measure amongst frail elderly people living in a residential care facility with compliant flooring. Through this, generate a theory that further explains the underlying barriers of active prevention amongst elderly people.

**Method**: We used the grounded theory method and conducted semi-structured in-depth interviews with eight elderly people in residential care (data collected between February and December 2017).

**Results**: The identified categories were *Falling as a part of life, Fearing the consequences* and *A wish to prevent falls and injuries*. Through the results it was clear that *There is more to life than risk avoidance*, permeated the interviews, therefore forming the grounded theory. The interviewees viewed falls as something common and normal, and were uninterested in focusing on the risk of falls. Although they wanted to prevent falls, it was often difficult to integrate preventative measures into their everyday life. They embraced the idea of an injury-reducing compliant flooring, however their main interests lay elsewhere, preferring to focus on social interaction and issues concerning daily activities.

**Conclusions**: The theory generated in this paper proposes explanations on the obstacles of implementing fall prevention measures in an elderly frail population. The findings give insights as to why interest and compliance for active fall prevention measures are low. We conclude that complaint flooring, from the perspective of the residents, can work well in residential care.

## Introduction

Falls are the most common cause of injury in all ages. Falls among older people are associated with increased morbidity and mortality, and decreased function (WHO, 2008). In Sweden, 70,000 people are annually admitted to hospital for fall-injuries (Socialstyrelsen, 2017), a majority older than 80 years of age. In addition, the older a person gets the more severe the injury tends to be (Grundstrom, Guse, & Layde, 2012) and elderly people in residential care are most at risk (Rapp et al., 2012).

**Figure 1.**
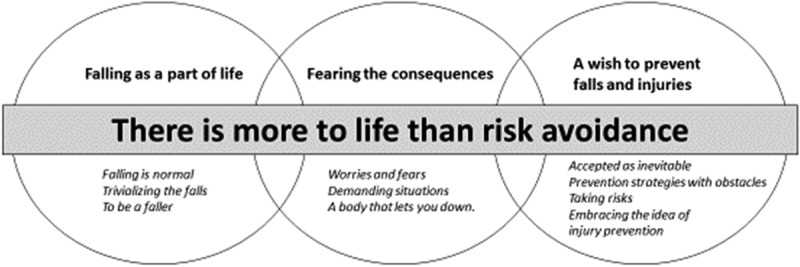
Theoretical model.

Despite considerable research, it has proven difficult to prevent falls and injuries among frail elderly. Preventative measures such as exercise, education and vitamin D supplements have been shown to have a non- or inconclusive effect in the residential care facility population (Avenell, Mak, & O’Connell, 2014; Bunn et al., 2014; Cameron et al., 2012). Although a review of complex multifactorial intervention programs has shown a reduction of recurrent fallers (Vlaeyen et al., 2015), there are considerable barriers for implementation outside the highly controlled circumstances of a study (Vlaeyen et al., 2017). As an alternative to active interventions, compliant flooring, defined as a flooring with some level of shock absorbency, has been suggested as a potential method for reducing the risk of fall-injuries among a frail elderly population (Lachance et al., 2017).

The overall research on elderly people’s views on the risk of falls, fall-injuries and potential prevention is scarce, especially regarding people living in residential care. However, previous research indicates that elderly are not particularly interested in talking about falls (Clancy, Balteskard, Perander, & Mahler, 2015). Considerable barriers among home dwelling elderly in participating in prevention programs has been identified, such as fatalism and an under-estimation of the risk of falling (Bunn, Dickinson, Barnett-Page, Mcinnes, & Horton, 2008; McInnes & Askie, 2004), taking precautions to prevent falls, due to poor health and functional ability, social awkwardness, and lack of motivation (Franco et al., 2015; McInnes, Seers, & Tutton, 2011). Given that compliant flooring requires no active participation from the elderly individual, the attitude towards the intervention could differ compared to other fall-injury prevention methods. Therefore, the aim of this study is to explore the experiences of falls, the risk of fall-injury, prevention in general and specifically compliant flooring as an injury preventative measure amongst frail elderly people living in a residential care facility with compliant flooring. Through this, the study aims to generate a theory that further explains the underlying barriers of active prevention amongst elderly people. However, the theory will not claim to present the full and final explanation. Rather, this study is an attempt to initiate an explanation to this complicated question, which can be refined by future findings.

## Method

In this qualitative interview study with elderly people, residing in a geriatric residential care unit with compliant flooring the overall design of grounded theory according to Glaser (1978); Glaser and Strauss (1967) was chosen. This is a method suitable to achieve a better understanding of a little-researched subject (Starrin, Dahlgren, Larsson, & Styrborn, 1997), and when a study aims to generate a theory explaining human behaviour (Glaser, 1978).

### Settings

The participants were recruited from two residential care units with compliant flooring, one in a rural area of Sweden and the other situated in a suburb near a larger city. To the authors knowledge, these are the only long-care residential units with compliant flooring in Sweden and both have used the same flooring product. The compliant flooring, marketed under the trademark Kradal, is a 12 mm thick, flexible composite tile (500 × 500 mm). All participants in this study lived in rooms with compliant flooring and in one of the wards it was also installed in the communal dining room and parts of corridor. The compliant flooring was not installed in bathrooms, as the flooring was not approved for wet areas.

The residential care units were part of a high care facility with access to 24-hour medical care as well as assistance with activities of daily live. Elderly in Sweden generally continue to live at home, even when having extensive care needs, due to the subsidized home care services. Residential care is only offered to elderly when they are deemed eligible according to the Swedish Social Services Act (SFS 2001:453). Therefore, elderly living in residential care in Sweden are a relatively homogenous group, often with considerable cognitive and/or physical impairments, requiring supervision, support and extensive care (Jensen, Lundin-Olsson, Nyberg, & Gustafson, 2002).

Due to its construction, the studied flooring was noticeably softer to walk on. One possible concern with using a different floor is that the elderly can experience differences when walking. The compliant flooring had previously been tested for balance impact, with no significant difference in gait stability found when compared to vinyl and carpet (Wright, Heckman, McIlroy, & Laing, 2014). It had also been tested concerning impact reduction during lateral falls on the pelvis, showing an overall protective effect, especially for frail elderly (Bhan, Levine, & Laing, 2013). We previously evaluated the injury reducing effect of the floor and found it to decrease the risk of injury by 59% for falls on compliant flooring compared to regular flooring (Gustavsson, Bonander, Andersson, & Nilson, 2015).

### Participants

We used a purposive sampling method (Palinkas et al., 2015) and the inclusion criteria were: individuals with sufficient cognitive ability for participating in an interview. They should also have lived in residential care with compliant flooring for at least three months, a reasonable time to settle in to the new situation and to have gained experiences of interacting with the flooring in different situations. Participants were recruited in cooperation with the staff at the two residential care units. The staff were informed about the aim of the study and the inclusion criteria for participation. They then identified suitable candidates, who were informed verbally and in writing so that they could make an informed decision whether to participate or not. During the data collection process, all potential participants, as identified by the staff, were asked to participate and they all gave their consent to take part in the study. The time of theoretical sampling was approximately 10 months (February-November 2017). The interviewees were between 74–94 years old (mean age 85) and both men and women were represented (2 men, 6 women). Some had mild cognitive impairments, an aspect that was exemplified by forgetting about agreeing to the interview, or not remembering the researcher when meeting the day after. Despite this, they could all tell a coherent story about how they came to live in the residential care home and had no problem answering questions. Two of the informants were in a wheelchair, others had a walker and one walked without mobility aid. None of them walked outside on their own. All lived in a single apartment, apart from one interviewee who lived together with a spouse.

### Data collection and analysis

The data collection and analyses were performed parallel using grounded theory (Glaser, 1978), during the time period February until December 2017. Data collection was performed using semi structured in-depth interviews (Kvale & Brinkmann, 2009). The interviews were carried out individually by one of the authors (JG) in the informants’ apartments at the residential care units. They lasted in average one hour and ten minutes each. JG has extensive experience of working with frail elderly and fall-injury prevention as a registered nurse, and has been involved in evaluating different effects of compliant flooring. FN, one of the co-authors, with a degree in physiotherapy and a PhD in risk management, has both practical and research experience of fall-prevention for elderly people and has been involved in evaluating other aspects of compliant flooring. FN and JG have visited the sites numerous times and made various observations regarding the flooring. CJ, with a PhD in public health, has not been directly involved in the evaluation, and her involvement could therefore help to control for preconceptions (Malterud, 2001). CJ also contributed with valuable experience of qualitative method.

A semi-structured interview guide was used to address the older person’s experiences and perceptions on the present subject; the risk of falls and fall-injuries, how they dealt with and prevented falls and injuries, and their understanding of compliant flooring and fall-injury prevention in general. The questions were open and related to different aspects of falls and fall prevention, e.g., –*please describe a fall that you have experienced*?, and followed up by in-depth questions and encouragements to clarify details (Charmaz, 2006). All interviews were tape-recorded and typewritten verbatim and data was analysed continually, parallel to data collection. The method of theoretical sampling was applied to guide the data collection. The initial open coding and memo-writing was done by JG. All three authors then participated in the continued analytical process, from focused coding to a conceptual level with theoretical coding specifying the relationship between categories. In order to reach theoretical saturation, the method of constant comparison was used, with the purpose of exploring variations, similarities and differences between the identified categories (Hallberg, 2006). These steps were all parts of the process that aimed to form a theory that is grounded in data (Glaser, 1992). Illuminating quotes are presented in the results to clarify how the empirical data supports the subcategories; quotes are marked with the number of the interview.

### Ethical considerations

The interviewees were frail elderly people and this called for special considerations in the interview situation. The interviewer tried to be attentive to the elderly person’s situation and needs. This could involve trying to create a relaxed atmosphere, adjusting the length of the interviews, finding a suitable time during the day when the person was alert, and not pressuring sensitive questions *(e.g., backing off when needed, and being observant on negative emotional reactions to questions)*. Most informants were interested in sharing their stories, even though they were puzzled about the aim of the study and did not think they had anything to say on the subject. None of them had been interviewed for research purposes before, and they had only a vague knowledge about what it entailed.

Ethical approval was received from the regional ethical board (Nr 2016/267) and the study was performed in accordance with the “Ethical guidelines for nursing research in the Nordic countries”.

## Results

The risk of falls and fall-injuries are constantly present in the elderly person’s life but even so, other things concerned them more. They preferred to talk about what gave them true meaning to life, such as their loved ones, people and activities that brought them joy and events in the past that had been of significance to them. They were generally happy and content with their lives and for many, the support and care provided in residential care had given them a richer life with new friends and access to meaningful activities. “*Somehow, they (the children) succeeded in persuading me. So I moved here. And I haven’t regretted that, I like it here. The children and I are close. We’re in contact 2–3 times a week” (3)*. Even if preventing falls and injuries was considered important, it was for various reasons not always prioritized. It could be that they chose to ignore the risks or that they were forced to take risks in a particular situation. When the participants were presented with the compliant flooring as a possibility to prevent injuries, the intervention was most welcomed, as they feared the consequences of falls. However, importantly, the risk of falls and fall-related injuries did not define their daily living, forming the theory: *There is more to life than risk avoidance*. The theory is presented throughout the result and is comprised of the following categories: 1. *Falling as a part of life*, 2. *Fearing the consequences*, and 3. *A wish to prevent injuries* (Figure 1).

### Falling as a part of life

The interviewees viewed falls as a part of life, something that they just had to learn to live with. In the process of normalizing the falls, they explained why they fell and diminished the importance of the events. This category, *falling as a part of life*, consisted of three subcategories: *falling is normal, trivializing the falls* and *to be a faller*.

#### Falling is normal

The risk of falls and fall-injuries were constantly present in the interviewees’ lives and they all had experience of falling. The falls were described as natural and normal, a part of everyday life, and an everyday event. Falling could even be something that they were so used to that they considered it to be a habit. Falls just happened. “*It’s just these little things (the falls), but otherwise it’s gone well and I haven’t broken any legs or arms or anything. They (the staff) have come and helped me up and I’ve carried on” (1)*. It seemed difficult for them to remember fall-related incidents, they would quickly change the subject, rather talking about other things, such as memories from their lives. This could be because of their cognitive inability, but also due to the ordinary nature of falls.

#### Trivializing the falls

When telling stories about fall-events, the interviewees tended to trivialize them. They talked about the falls as nothing special, just a minor event. Sometimes they did not even want to admit that it had happened. *I don’t know, I fell on to the floor. There’s not much more to say about it. (2.2)*. They also diminished the importance of the falls by emphasizing that most often there was no serious consequence. Even if the fall resulted in a serious injury, they were described in an unsentimental and direct manner. “*I was in another care home and I fell. I hit my head badly and was bleeding, but that’s common” (3)*.

#### To be a faller

In some of the interviews, being a faller was almost a part of how the interviewees viewed themselves, i.e., a part of their identity. One women had experienced falls throughout her life, and it was something that she had always had to deal with and relate to. To identify oneself as a person that falls could be a way of explaining why you fell. Conversely, when viewing oneself as a physically capable person, a fall could be quite the surprise and something that you are unwilling to internalize.

### Fearing the consequences

Although falling was seen as a part of life, there was also a fear of the consequences and the feeling that the body no longer could be trusted. This category, *fearing the consequences*, consisted of three subcategories: *worries and fears, demanding situations*, and *a body that lets you down.*

#### Worries and fears

The interviewee’s biggest concern was not the risk of falling, it was more in the line of concerns for others and that something should happen to their loved ones. However, they were well aware that a fall could have serious consequences and they feared that they would lose skills and abilities, end-up bedridden or worst case, that it could mean the end of their lives. “*The fall can be the end of me. I once saw someone fall by the toilet door and they never recovered. It makes you think about the consequences of a fall.” (1).*

Some had first-hand experience; others had seen someone close to them be seriously injured. In addition to the risk of physical injuries, the interviewees brought up psychological aspects of falls. Falls were seen as shameful or embarrassing, for example if a fall resulted in an unnecessary visit to hospital. It could also be important to not show pain despite being hurt, reassuring staff and relatives that an injury was less serious or less painful. This also involved the residents’ wishes to not worry their relatives. For example, one woman described a situation where she had fallen and didn’t want her son to visit her since she was concerned that he would get worried if he saw her.

#### Demanding situations

Being in a new environment could lead to a decline in self-confidence, causing anxiety that could lead to a fear of walking by oneself. In addition, social events were described as demanding and sometimes anxiety filled, despite also being important life-fulfilling activities. For example, one woman talked about a birthday party in which the situation in itself made her feel insecure and affected her ability to walk.

#### A body that lets you down

The fear of falling and the risk of falling was often linked to a decline in physical functions. Medical problems could be used to explain an increased risk of falling, and claiming medical reasons was a way to find explanations to the fall. The interviewees described that bodily functions were not to be trusted anymore, for example legs were weak, a loss of general strength or impaired motor skills. This could be due to medical problems such as cardiovascular disease, or issues of a more degenerative nature. One woman expressed that her knees no longer could be trusted and that she was afraid of walking and scared to death of falling.

### A wish to prevent injuries

All interviewees agreed that preventing falls was very important as falls could lead to serious consequences. They were therefore very grateful for the preventative possibilities that were available. However, prevention was generally difficult and there were other aspects that would overshadow fall risks and were seen as more important than avoiding falls. The category, *a wish to prevent injuries*, consisted of four subcategories: *Accepted as inevitable, Prevention strategies with obstacles, Taking risks* and *Embracing the idea of injury prevention.*

#### Accepted as inevitable

All interviewees clearly pronounced that they fell because the falls were inevitable and they fell unexpectedly, and without explanation. The fact that falls were viewed as inevitable emphasized the notion that they were also unpreventable. One interviewee expressed that she was not afraid of falling since there was no point in being afraid since nothing can be done to prevent it. “*You can’t prepare for them (the falls), it never happens at the same time. When it happens it happens. It’s simply a surprise” (3)*. Some talked about getting a hunch that something was about to happen, but even then, they were often not able to prevent the fall. Even though the interviewees believed the floor to be protective, they did not see the risk of fall-injury as being eliminated, due to the inevitable nature of the falls, and the fact that they could be injured on other objects than the flooring.

#### Prevention strategies with obstacles

As mentioned previously, other aspects of life were often seen as more important than fall prevention. The ideas to prevent falls were often vague and generalized. “*I just think, I shouldn’t fall”* (*1)*. Trying to be careful and holding onto things when walking was a common method to try to protect oneself from falling. “*I think you have to be very careful. When I’m going to sit here on this chair, I’ll take small steps and turn around. I’m very, very careful of course” (1)*. There was an awareness of an increased risk when climbing stairs and either human or physical support was often used to decrease the risk. Others had clearer strategies of how to avoid or to lower risk. To ask for help, especially with more risk-filled tasks such as hanging curtains, was one example. Mobility aides, such as walkers, were considered to protect from falls, and were seen as important to have nearby at all times.

Medical treatments that reduced the risk of falling were appreciated, however, some medication could also contribute to the risk of falling. Some pills made them dizzy and confused and there were vivid descriptions of various unpleasant events such as hallucinations. “*The nurse said I could take them three times a day. I did, but I got dizzy and unsteady” (1)*. An awareness of the risks of taking medications made the interviewees attempt to find a balance between the need to reduce pain and the increased fall risk associated with the medication.

#### Taking risks

Despite being aware of the risk of falls, as well as the injuries that could follow, there were situations where the interviewees choose, or felt forced, to put themselves at risk. Avoiding risks could come into conflict with other aspects that were more important, such as preserving one’s autonomy or independence and doing things that they wanted. Also, more mundane, acute needs, such as needing the bathroom or a lack of access to the alarm, lead them to taking risks. Not wanting to disturb the staff was another component, sometimes combined with an element of defiance.

There were also situations when mobility aides worked poorly or were too complicated to use, leading to risky behaviour. For example, a wheelchair that was perceived as unwieldy, or a walker that was difficult to manoeuver in the bathroom.

#### Embracing the idea of injury prevention

The idea of a compliant flooring as a means to protect from injury was greatly appreciated. “*It’s very good if it (the floor) prevents injuries if you fall. It’s so difficult to recover after an accident”* (4). They were grateful for the protective floor for their own sake, but also because they worried friends and loved ones would fall and injure themselves. A floor that protects from injury was also something that they could imagine having in a private home as well. Despite being generally positive, there were some concerns. For example, the quality of the flooring didn’t match their expectations and there were some problems with mobility aides, hoists and wheelchairs due to the softer surface. When walking with mobility aides, for example, the wheels tended to get stuck making it harder to turn. However, others thought that whilst it was heavier to push a wheelchair, the difference wasn’t significant. One interviewee felt that she had been forced to move in to residential care and had no opinion of the special flooring; except that she had little faith in its protective capacities. For her the special flooring was yet another cause of frustration in an unwanted living situation.

Finally, another concern was that the flooring could have a negative effect on the staff. The wellbeing of the staff was very important to them and someone had noticed that it was harder to move equipment, for example beds, and worried that this would be a strain on the staff.

The interviewees wanted the compliant floor to be protective and their thoughts on the effectiveness of the compliant flooring were largely based upon what they’d heard, their own experiences of falling on it, and/or seeing others falling on it. One woman said that it was soft to fall on; another had heard that the compliant flooring protected against injuries but didn’t really understand how it worked. “*For me, it’s very important (the floor). Very important to me considering how much I’ve fallen before. Important to have a floor that can protect me from injuries. So it’s a top priority. You’re protected so you don’t break anything” (3)*. The notion that the flooring in general was a good intervention seemed to be based on the wish to prevent fall-injuries, and the idea of a floor that protects was of considerable importance for an elderly person with an experience of falls and injuries. Most didn’t feel or notice any difference when walking on various floor surfaces although the quietening component of the flooring was appreciated by some.

## Discussion

This qualitative interview study describes frail elderly people’s views and experiences on various aspects of falls, fall-injuries and prevention. A key finding was that the concept of falls seems to be a fully integrated part of life for elderly people living in residential care. They talk about falls as something normal and find logical ways of rationalizing the causes behind their falls, such as physical decline or falls being accidents beyond their control. This is supported by previous research showing that an experience of falls does not necessarily mean that falls become an important part of the elderly persons identity (McInnes et al., 2011). When objectively observing a frail elderly person it may seem as if their quality of life is low, given that they are often alone and suffer from different forms of impairment. However, this is not necessarily the case. When asking elderly people it is clear that they often enjoy life and experience great satisfaction (Tornstam, 2011).

Simultaneously, there was an apparent awareness of the risk of serious consequences of falls and this was something that they feared. The fear was primarily concerned with the risk of losing abilities and becoming dependent upon others. Whilst fear of falling has been shown to increase the risk of falls (Hadjistavropoulos, Delbaere, & Fitzgerald, 2011), the results in this study show little signs that fear greatly affects their lives. Rather, the results supports previous research showing that an awareness of risk does not necessarily corresponds to an active consideration of preventative measures (McInnes et al., 2011).

The participants in this study say that they wish to prevent falls and fall-injuries but at the same time other things seems to be more important. These aspects of life can be regular activities of daily living that make them forget or ignore taking precautions, but also social and emotional factors that overshadow the desire to adapt. Preserving one’s identity and independence has previously been shown to be more important to an elderly person than adapting one’s life to the risk of falling (Clancy et al., 2015; McInnes et al., 2011).

This relative lack of interest, or prioritization, for fall prevention creates obvious problems in the implementation of preset fall prevention programs especially with regards to compliance. For example, participation in programs for physical activity is generally low (Franco et al., 2015), few elderly people want to make home modifications when these are proposed by health care personal (Turner et al., 2011) and despite evidence of hip protectors having an injury reducing effect, compliance is low (Santesso, Carrasco‐Labra, & Brignardello‐Petersen, 2014). Even if evidence points towards a lack of interest in prevention, the participants in this study do take precautions. They mention asking for help, using mobility aides and being careful as means to prevent falls. There is evidence that prevention is more likely to be successful if designed on the persons own terms. For example, to be able to live the life that they want, elderly can be willing to make adjustments in their homes and organize their lives according to the present circumstances (Barker, Morello et al., 2016, Mahler, Sarvimäki, 2010). There are indications that instead of focusing on the risk of falling, they tend to adjust to a life according to the present circumstances and to make preventative measures on their own terms (Kruse, Moore et al., 2010). The physical environment has the potential to support the elderly person, an example is that furniture can be organized so that it supports movement and that there is room for mobility aides (Mahler & Sarvimäki, 2012).

In addition to the physical consequences of falls, there could be feelings of shame and embarrassment connected to falls and injuries. This aspect has also been seen in previous research (Host, Hendriksen et al., 2011) and something that a compliant flooring does not seem to mitigate, i.e. it seems to be equally shameful to fall on a soft surface as a hard surface. However, the stigma that might follow with having to use other more noticeable preventative strategies, such as a walker, and thereby potentially threaten to change the elderly person’s sense of self, is not the same for compliant floor.

The theory of socio-emotional selectivity states that time is an important factor for how we make health decisions (Löckenhoff & Carstensen, 2004). The more time a person perceives to have left in life, the more they tend to make decisions according to long time goals and engage in activities that benefit learning and that are potentially rewarding in the future. A person that instead perceives that they have limited time left in their life tends to be more interested in activities that are instantly emotionally rewarding and is less likely to engage in activities that generate long-term benefits. The theory on socio-emotional selectivity could therefore support the increased use of fully passive interventions for very frail elderly people. Compliant flooring is a technical, structurally integrated intervention requiring no compliance or active decision-making from the elderly person themselves. Given the results in this study, it could be argued that interventions such as compliant flooring are highly relevant and well-suited for a population where falls and fall-injuries are feared, although injury prevention is less prioritized compared to other aspects in life.

### Method discussion

The theory formed in this study is an attempt to explain the frail elderly person’s perspectives on the concept of falls and the underlying barriers of active prevention in this population. The quality of a grounded theory depends on several aspects; the theory must fit the data, be relevant, work to explain and predict the studied phenomenon, and be modifiable to changes (Glaser & Strauss, 1967). In an attempt to meet these criteria, we did not use any predefined categories or theories, the data collection and analytical processes were intertwined, guided by theoretical sampling, and performed with a constant comparison of identified categories.

The main limitation of this study is the question of theoretical saturation. Many individuals in Swedish residential care have considerable cognitive disabilities and in order to collect relevant data, the staff at the facilities helped to identify persons that were cognitively able to participate, thereby leading to eight interviews. Whilst it could be argued that more interviews were needed, reaching saturation in grounded theory is a complex process, including more than merely the number of interviews. Other aspects of importance are the nature of the question, the researcher’s level of experience in qualitative research, the philosophical understanding of the method, and the use of a guiding theoretical framework (Aldiabat & Navenec, 2018). Using the recommended procedures mention above assists saturation. There are also factors that can hinder reaching theoretical saturation, such as conducting the research within a short period of time, having a limited budget for conducting the research work, and limited resources regarding training and monitoring (Aldiabat & Navenec, 2018). We claim that our research question is relatively direct and that we had the relevant experience to understand the studied phenomena, something that can facilitate saturation. In addition, the process of data collection and analysis was allowed to take time and the first author requested directions from the other more experienced authors throughout the process. Based on the nature of our data, we argue that we reached a relatively good level of saturation, despite the limited number of interviews. The concepts, and linkage between concepts are verified, an important marker for theoretical saturation. Simultaneously, however, the theory should be open to future modification if new information appears. Therefore, full saturation in grounded theory is a complicated concept.

## Conclusions

The results in this paper propose explanations on the obstacles of implementing active fall prevention in an elderly frail population. The interviewees are well aware of the risk of falls, they are afraid of the consequences that a fall can have, and they are interested in preventing falls. Despite this, these aspects play a relatively unimportant role in their lives and do not affect them to a substantial degree. Rather, their main interest lies elsewhere. These findings can give us insights as to why interest and compliance for active fall prevention measures are low. Compliant flooring is a passive fall injury measure that does not require the target group to make decisions, adapt or actively participate in the program and the participants appreciate this potentially protective capacity of compliant flooring. Therefore, although there were some negative issues raised, we conclude that complaint flooring, from the perspective of the residents, can work well in residential care.
